# Refinement of a scoping tool to tackle antimicrobial resistance via a primary health care approach: insights from Kazakhstan, Sweden, and Thailand

**DOI:** 10.1016/j.lanprc.2026.100211

**Published:** 2026-09

**Authors:** Serena W Y Chong, Sarah Paulin, Faraz Khalid, Stephan Stenmark, Danilo Lo Fo Wong, Deborah Tong, Ketevan Kandelaki, Viroj Tangcharoensathien, Bassem Zayed, Abzal Zhumagaliuly, Erica Barbazza, Shamsuzzoha B Syed, Nienke Bruinsma

**Affiliations:** a Antimicrobial Resistance Department, WHO, Geneva, Switzerland; b Department of Performance, Financing and Delivery, World Health Organization, Geneva, Switzerland; c Department of Communicable Disease Control and Preparedness, Public Health Agency of Sweden, Stockholm, Sweden; d WHO Regional Office for Europe, Copenhagen, Denmark; e Science and Programmes Department, International Centre for Antimicrobial Resistance Solutions, Copenhagen, Denmark; f Pandemic Threats, Communicable Diseases and Antimicrobial Resistance Unit, WHO Regional Office for Europe, Copenhagen, Denmark; g International Health Policy Program Foundation, Nonthaburi, Thailand; h Department of Health Systems, WHO Regional Office for South-East Asia, New Delhi, India; i B Atchabarov Scientific Research Institute of Fundamental and Applied Medicine, Asfendiyarov Kazakh National Medical University, Almaty, Kazakhstan

## Abstract

WHO recommends a primary health care (PHC)-oriented health system for delivering high-quality, equitable, and efficient care in all countries. Primary care and the community serve as the frontlines for preventing and managing infections and addressing the rise of antimicrobial resistance (AMR). WHO developed a scoping tool to identify AMR-relevant interventions that can be used to strengthen PHC-oriented health systems and applied it to country reviews in Kazakhstan, Sweden, and Thailand. The objective was to identify AMR interventions in primary care and the community that are context relevant, improve quality of care, and strengthen health systems. These interventions included the integration of AMR-related efforts, antimicrobial stewardship, and infection prevention and control into health system policies, practice, and governance; promotion of appropriate antimicrobial use, including use of routine health data; stronger pre-service education and in-service training and support for the primary care workforce; and ensuring access to essential medicines while enforcing regulations on non-prescription sales of antibiotics. Foundational elements of health systems such as water, sanitation, and hygiene and immunisation programmes were also essential for a comprehensive AMR response. This process highlighted the need to engage with a diverse range of stakeholders and to consider social and contextual differences when designing AMR-relevant interventions. National AMR programmes should collaborate with health-system planners to integrate AMR-related interventions into PHC-oriented health systems, strengthening them to drive progress in countries towards universal health coverage while achieving global targets on AMR.

## Introduction

In 2019, WHO identified antimicrobial resistance (AMR) as one of the top threats to health and health systems globally.^1^ As antibiotics and other antimicrobials become ineffective owing to the rise in AMR, the ability to treat infections and perform routine medical procedures is compromised. The GBD 2021 Antimicrobial Resistance Collaborators estimated that in 2021, 4·71 million deaths worldwide were associated with bacterial AMR, of which approximately 1·14 million were attributable to bacterial AMR.^2^ The World Organisation for Animal Health and The World Bank have projected that health-care costs of AMR will rise to US$159 billion per year by 2050 if resistance rates follow historical trends since 1990.^3^ Although the development of resistance is a natural process, inappropriate and overuse of antibiotics can greatly accelerate the emergence and spread of bacterial resistance. To decrease the burden of AMR in health systems worldwide, three areas for action are key: first, preventing infections; second, accurately diagnosing infections; and third, ensuring that antibiotics are used appropriately. Health systems therefore need to be well equipped to implement these interventions.

Most national AMR surveillance data come from secondary and tertiary care facilities.^4^ Given that these facilities are more likely to have patients with severe infections, including health-care-associated infections and those caused by drug-resistant pathogens, these data are often not representative of the situation in primary care facilities.^5^ Antimicrobial stewardship (AMS) programmes, a cornerstone of AMR management, have also historically been implemented in secondary and tertiary care, and primary care professionals are often not the focus of AMS capacity-strengthening efforts. However, most patient interactions occur at the primary care level and antibiotics are often dispensed in primary care and the community (ie, 85–95% of total antibiotic use in the community in 2015).^6^ In low-income and middle-income countries (LMICs), inadequate access to essential medicines and diagnostics or, conversely, unrestricted over-the-counter sales of antibiotics and suboptimal prescribing practices, continue to contribute to high levels of inappropriate antibiotic use.^7^ This problem is particularly true for antibiotics classified in the Watch category of the WHO Access, Watch, and Reserve (AWaRe) classification,^8^ which made up 45% of global antibiotic use in 2022.^5^ Watch antibiotics include broad-spectrum antibiotics such as third-generation cephalosporins, carbapenems, and fluoroquinolones, exposure to which likely increases the risk of colonization or infection with multidrug-resistant organisms.^9^

Primary care professionals need to make first decisions regarding the appropriate diagnosis and treatment of infections (including drug-resistant infections), often amid diagnostic uncertainty, which can be due to a lack of point-of-care diagnostics or decision-support systems, and under pressure because of time constraints and patient expectations to receive a prescription. In many settings, non-prescription or over-the-counter access to antimicrobials, limited infection prevention and control (IPC) capacity, and gaps in workforce competencies compound these challenges.^10^ Therefore, strengthening AMR action in primary care is not only a policy question but also a practical one for frontline health-care professionals; the workforce and systems around these professionals need to be equipped to prevent, diagnose, and treat infections appropriately. The loss of effective first-line antibiotics for common conditions will also be most keenly felt in primary care. Therefore, primary care and the community should be central to the AMR response effort as they are the foundation of effective and efficient health systems, with evidence supporting their contribution to reduced mortality and morbidity, improved health equity, and enhanced trust in health-care systems.^11^

In 2024, WHO Member States endorsed the WHO strategic and operational priorities for 2025–35 to address drug-resistant bacterial infections, which called for a people-centred approach to AMR (PCA-AMR) and embedding AMR interventions within health-system-strengthening efforts.^12^ This approach is reinforced in the Global Action Plan on AMR 2026–36.^13^ WHO’s PCA-AMR consists of a core package of 13 interventions to be implemented at all levels of care.^14^ To support countries in operationalising these core interventions at the primary care and community levels, WHO developed an AMR and PHC scoping tool. This tool identifies AMR-relevant actions that can be strengthened and integrated into PHC-oriented health systems according to the three components of the WHO–UNICEF operational framework for PHC:^15^ multisectoral policy and action, empowered people and communities, and integrated health services, with an emphasis on primary care and essential public health functions.^15^ In this Health Policy paper, we consider all three components of this PHC operational framework, encompassing, but not limited to, primary care, which forms the core of integrated health services.

WHO worked closely with Kazakhstan, Sweden, and Thailand in 2024 and 2025 to refine and finalise the scoping tool. Key findings and recommendations from the three countries were synthesised to identify priority areas for these countries to focus on when planning and implementing AMR-relevant actions in primary care and the community.^16^ Here, we describe the methodology and results from the scoping tool refinement process, including country-specific recommendations arising from the country reviews in Kazakhstan, Sweden, and Thailand, and cross-country findings that informed the development of a WHO policy brief, *Strengthening Primary Health Care-oriented Health Systems to Address Antimicrobial Resistance*.^16^

## Methods

### AMR and PHC scoping tool

The WHO PCA-AMR core package of 13 interventions suggests priority actions at national, secondary or tertiary care, primary care, and community levels. To support countries, WHO developed a scoping tool to operationalise the priority actions at the primary care and community levels using guiding questions and considerations with inputs from WHO experts and Collaborating Centres working in the AMR and PHC domains. A preliminary version of the scoping tool contained 26 focus areas and indicators, grouped by the three components of PHC as captured in the WHO–UNICEF operational framework for PHC^15^ and the accompanying PHC measurement framework and indicators:^17^ (1) multisectoral policy and action; (2) empowered people and communities; and (3) integrated health services with an emphasis on primary care and essential public health functions. WHO refined the scoping tool on the basis of observations and findings in the three countries and drawing on lessons learned from country priorities, resulting in 21 focus areas and indicators (table 1) in the final scoping tool, which has been published in a policy brief.^16^

**Table 1:** The 21 focus areas of the AMR-PHC scoping tool grouped by the three PHC components and those selected by countries

	Kazakhstan	Sweden	Thailand
Multisectoral policy and action			
1. Integration of AMR into national PHC policies and strategies	✓	✓	✓
2. Mechanisms for governance of AMR, PHC, and universal health care	✓	..	✓
3. Inclusion of civil society and professional associations in defining AMR response	✓	..	..
4. Engagement of the private sector in AMR response	..	..	..
5. Policies integrating appropriate antimicrobial use and AMS principles and activities in primary care∗	..	✓	✓
6. Regulations to restrict non-prescription sales of antimicrobials	✓	..	✓
Empowered people and communities			
7. Community health promotion on AMR	✓	✓	..
8. Community-based systems for the return of unused antimicrobials	..	..	..
9. Community engagement in access to high-quality AMR-related products, antibiotics, and services	✓	✓	..
Integrated health services with an emphasis on primary care and essential public health functions			
10. Pre-service education on AMR for primary care workers, including community health workers and community pharmacists	✓	..	✓
11. In-service training and support on AMR for primary care workers, including community health workers and community pharmacists	✓	..	✓
12. Primary care facilities that contribute data to AMR surveillance systems	..	..	..
13. Surveillance of antimicrobial use in primary care facilities and community pharmacies	..	✓	..
14. Water, sanitation, and hygiene in primary care facilities	..	..	..
15. Infection prevention and control in primary care facilities	..	✓	✓
16. Immunisation	..	..	..
17. Affordability of essential medicines and health products, including vaccines, diagnostics, and antibiotics	..	..	..
18. Availability of essential medicines, including essential access antibiotics, in primary care facilities	..	✓	..
19. Availability of essential diagnostics for pathogen identification and antimicrobial susceptibility testing in primary care facilities	..	..	..
20. Appropriate treatment ensured by AMS in primary care facilities∗	..	✓	..
21. Adoption of the WHO AWaRe Antibiotic Book to guide appropriate treatment in primary care facilities	..	..	..

AMR=antimicrobial resistance. AMS=antimicrobial stewardship. AWaRe=Access, Watch, and Reserve. PHC=primary health care.

∗ Although item 20 is conceptually related to item 5, it explicitly considers whether AMS activities and principles are implemented in clinical practice; item 5 refers to a more general consideration of whether AMS policies and guidelines are available, such as clinical treatment guidelines for primary care.

### Country selection

WHO worked with three selected countries—Kazakhstan, Sweden, and Thailand—for reviews, as they represented diverse national income levels, PHC structures, and cultural contexts. In 2024, Kazakhstan and Thailand were both upper-middle-income countries and Sweden a high-income country according to The World Bank.^18^ This selection of countries allowed for the relevance and adaptability of the scoping tool to be tested across different health system settings. The process was also subject to time and geographical parameters because of funding source requirements. Country readiness to undertake this work was also considered owing to time constraints.

In each country, WHO identified national focal points on AMR and PHC from ministries or public health agencies to support reviews, which included a desk review and country mission. WHO, with support from national focal points, used the preliminary AMR and PHC scoping tool to take stock of the country’s AMR-relevant actions in PHC, selecting six to ten focus areas per country for an in-depth review based on their context, with consideration of gaps and opportunities to accelerate action on AMR and PHC, or where work was already in progress.

### Data collection

WHO collected preliminary evidence on each of the selected focus areas in these countries through a desk review of primarily qualitative data and information. Data sources included published literature, national policy documents, government reports (in English, Thai, Russian, and Swedish), unpublished data provided by the country or WHO sources, and, in some cases, direct qualitative inputs from national focal points spearheading work on AMR and PHC in the country. Subsequently, guiding questions for each country were formulated on the basis of the preliminary evidence and evidence gaps identified (many of these guiding questions were also refined and eventually published as part of the scoping tool). WHO then organised a 3–4-day country mission to verify the preliminary evidence collected through the desk review, in close collaboration with the Asia-Europe Foundation and national counterparts—the Ministry of Healthcare in Kazakhstan, the Public Health Agency of Sweden, and the International Health Policy Program Foundation in Thailand. The country missions included a 1-day site visit to observe existing practices in select public and private primary care facilities and a 2–2·5-day national workshop with around 30–40 stakeholders on areas relevant to AMR and PHC; representatives from regulatory agencies, professional associations, and academia; and subnational PHC managers and administrators and health practitioners (table 2). Thus, primary data collected included observations from site visits and discussion outputs generated participatorily from the national workshops.

**Table 2:** Number of participants at the national workshops and PHC sites visited

	Number of participants at the national workshop	PHC sites visited
Kazakhstan	41	Four (two polyclinics and two hospitals providing primary care)
Sweden	27	Three (two primary care facilities and one community pharmacy)
Thailand	38	Six (two district hospitals, two subdistrict health centres, one private clinic, and one community pharmacy)

PHC=primary health care.

During the workshop, stakeholders discussed evidence, practices, and opportunities with respect to the focus areas selected and the guiding questions formulated as part of the desk review. The site visits allowed for observation of context and current practices.

### Data analysis

WHO triangulated the information gathered from desk reviews and country missions (ie, primarily qualitative information derived from field notes of site visits and stakeholder-generated information from workshop discussions) to identify country-specific entry points and recommended actions, which were published in country reports.^19^, ^20^, ^21^

Using elements of deductive content analysis, WHO further synthesised and clustered findings from the three countries to identify overarching themes that recurred across countries and were supported by the available evidence; this process identified eight priority AMR-relevant interventions in primary care and the community. These interventions, accompanied by non-country-specific examples and the final scoping tool, have been published in the WHO policy brief on AMR and PHC,^16^ while the country-specific and cross-country findings that form the basis for the priority interventions are described in this paper.

## Results

The 21 interventions of the final scoping tool grouped by the three PHC components and the focus areas selected by the three countries for the country reviews are shown in table 1, highlighting that the countries had some common priorities.

The country reviews resulted in mission recommendations published in the respective country reports.^19^, ^20^, ^21^ These reviews also informed the formulation of eight AMR priority interventions, contained in the WHO policy brief.^16^ The country results and evidence informing each of the priority interventions are detailed in the following sections.

### Multisectoral policy and action

#### Embed AMR-related interventions into national PHC-oriented policies, strategies, and related governance mechanisms

The country reviews highlighted that many of the existing quality-of-care practices in health systems are also AMR relevant because they prevent the spread of infections or promote appropriate use of antibiotics through optimised diagnosis and treatment. The practices might not be explicitly included in AMR national action plans or AMR-specific policies, or necessarily have the same terminology, although they are captured in relevant national health systems or policies and strategies (eg, on universal health coverage [UHC] or PHC) and could be funded through different parts of the health system.

For example, IPC was recognised as a priority in all three countries for preventing and minimising the spread of infections in primary care and the community and was reflected in national IPC policies and implementation of interventions in primary care facilities. Identified gaps included the availability of IPC-monitoring systems and use of data in primary care, which are areas for further strengthening. In Thailand, for instance, although national estimates are available for water, sanitation, and hygiene (WASH) in households and health-care facilities,^22^ a mission recommendation was to include more primary care facilities in national IPC assessments to better monitor and assess the effectiveness of IPC measures.

Recognising the need for multisectoral collaboration to comprehensively address AMR, all three countries established intersectoral working groups or committees to steer national-level policies and strategies on AMR. The reviews highlighted that a wide range of stakeholders need to be involved to enable sustainable and equitable implementation of AMR interventions and integration into broader health system-strengthening efforts, including through a PHC approach.

The stakeholders include non-traditional stakeholders, such as agencies or bodies with responsibility for public spending, education, social affairs, and accreditation, and others whose work directly affects many social determinants of health.^23^ Subregional authorities and professional associations also play a pivotal role in helping to translate policy into practice in primary care, especially in countries with decentralised health systems. Because the private sector increasingly provides a large proportion of health services in many countries,^24^ private health service providers need to be engaged to achieve necessary coverage on matters ranging from minimum quality standards for IPC and AMS to ensuring equitable access to quality-assured essential antibiotics and alignment of patient and laboratory data. Finally, civil society and community representation in policy and coordination bodies are often neglected. For instance, in Thailand, the mission recommended including local governments, civil society, and private sector representatives in the AMR coordination mechanism.

#### Stepwise enforcement of regulations to restrict non-prescription sales of antimicrobials

To address AMR in human health, WHO recommends regulating the sale of antimicrobials.^14^ However, regulations alone are insufficient to achieve adequate change, unless accompanied by prescriber, dispenser, and public compliance to, or enforcement of, regulations by authorities.

Kazakhstan, Sweden, and Thailand all have regulations governing the sale of antibiotics without prescription to varying degrees, with higher levels of compliance observed when regulations are supported by interventions such as public health education and pre-service education for health-care providers, as seen in Sweden^25^^,^^26^ and indicated in a recent study of LMIC data.^27^ In Kazakhstan, the mission recommended integrating the WHO AWaRe classification of antibiotics into regulatory documents and strengthening enforcement of restrictions on over-the-counter sales of antibiotics without prescriptions, starting with the Watch and Reserve groups of antibiotics. Similar mission recommendations from Thailand focused on the ongoing staged process of reclassification of antibiotics as prescription-only medicines while also considering the AWaRe classification,^28^ supplemented by extending pre-service education and mandatory in-service training as pharmacist-relicensing requirements to support appropriate dispensing of antibiotics.

#### Ensure access to essential medicines, including essential antibiotics

In all three countries, access to essential antibiotics emerged as an overarching theme even when not explicitly selected as a focus area. Each country faced different challenges. In Sweden, as in other Nordic countries, some off-patent antibiotics are not lucrative for manufacturers and importers, thus affecting their availability in the market.^29^ Global shortages of specific narrow-spectrum Access antibiotics, such as amoxicillin and amoxicillin with clavulanic acid, have also affected Sweden. These challenges resulted in prescribing complications for prescribers and dispensers and delays in patients receiving appropriate antibiotics. The Swedish Government is actively working on policy and interventions to ensure access to essential medicines, especially for generic antibiotics, including Access antibiotics typically used in primary care.^30^

In Kazakhstan and Thailand, the inclusion or exclusion of specific Access antibiotics in national treatment guidelines could have also affected the clinical demand and consequently the supply of those antibiotics. Interventions prioritised by national stakeholders in these countries were to introduce or expand the use of the WHO AWaRe classification in national medicine lists or formularies and use the WHO AWaRe Antibiotic Book as a resource to update national treatment guidelines, particularly for common infectious syndromes. These policy-level interventions can be connected to education and training to support the implementation of guidelines and balance access and excess.

### Empowered people and communities

#### Broad-based community health promotion on infection prevention and response to drug-resistant infections, supplemented by targeted engagement with vulnerable population groups

Public health literacy and health promotion have been used to influence behaviours regarding preventing and managing infections in Sweden and Thailand, including obtaining and using antibiotics appropriately.

Studies have suggested that Sweden’s success in addressing AMR could be due to high levels of health literacy and awareness in the population, including on antibiotics.^26^ This effect has been the result of decades of work to educate the public and continues to be supported by various ways of ensuring that individuals have access to necessary information in their language and increasing general community awareness through social media, the internet, and more recently, an initiative called Antibiotic Smart Sweden, which has enabled establishments such as preschools and hospitals to be declared as being antibiotic smart.^31^

In Thailand, district hospitals, which are a linchpin of the PHC system in the country, support and manage a network of community volunteers, also known as village health volunteers, who are part of their communities and therefore play an important role in transmitting health education and information. In Sweden, civil society groups, such as Transkulturellt Centrum, advocate on behalf of new immigrants and engage with local and subnational authorities on health promotion efforts. Additionally, in Sweden, materials on the health system and, specifically, on antibiotics were delivered as part of civil ethics courses that are mandatory for new immigrants and organised by regional authorities.

Alongside civil society involvement, other opportunities could exist to facilitate community engagement in a PHC-oriented health system. Community health workers or community pharmacists, which are both part of a PHC-oriented health system, serve as an interface between facility-based health care and a range of population groups in the countries. Professional associations also support the dissemination of knowledge and information in communities, as shown in all three countries.

### Integrated health services with an emphasis on primary care and essential public health functions

#### Promote appropriate use of antimicrobials

Even when AMS was not explicitly selected as a focus area for country reviews, it emerged as an important theme across discussions in all three countries. Cultural and language contexts shaped the understanding and practice of AMS principles, and many AMS principles were also linked to quality-of-care initiatives, such as rational drug use programmes.

Both Sweden and Thailand provided evidence that the use of incentives to promote appropriate antibiotic use practices can be effective. Thailand translated rational drug use policy into key performance indicators on the use of antibiotics for three common conditions (upper respiratory infection, acute diarrhoea, and fresh wounds) typically seen in primary care. In Sweden, AMS policies were defined nationally; implementation at the primary care level was actively supported by national and regional Swedish Strategic Programme Against Antibiotic Resistance (STRAMA) groups^26^ and incentivised by regional health authorities.

In both Kazakhstan and Sweden, the policy-level integration of AMS with IPC was prioritised. A recommended action in Kazakhstan was to review and develop indicators for AMS and IPC activities in primary care facilities. In Sweden, the recommendation was to include IPC and AMS quality indicators in so-called regional rule books that outline rules and responsibilities around health care.

#### Health information systems to support antimicrobial use surveillance and policies

Reviews from the three countries identified how routine health information and data could be used to support AMR-relevant interventions and strengthen quality of care. In Kazakhstan, public health facilities were digitalised and most facilities relied on electronic patient records. National-level antimicrobial sales data were available, which acted as a proxy for antimicrobial use, and the Professional Association of Clinical Pharmacologists and Pharmacists used national antimicrobial sales data for some analysis and reporting, also publishing and submitting the data to regional databases in 2025.^32^ Because large amounts of data are being generated, this opportunity can be used to promote data standardisation and use, for example, by aligning the types of prescription and diagnosis data collected between different health-care providers to facilitate the analysis and use of the data to inform prescriber and policy decisions.

In Thailand, most public health facilities had facility-level data on antimicrobial use that had been generated through an electronic patient records system. Efforts were made to analyse and publish the data at the national level, with an opportunity to increase the accessibility and use of data across different health-care providers, for example, to inform treatment decisions or update treatment guidelines.

The Swedish national electronic prescription database and data from electronic patient records facilitated the analysis and use of benchmarked prescribing and diagnosis data, linked to near real-time sales data, to inform many AMS activities conducted by STRAMA groups in primary care, including outreach, education, and prescriber feedback.^19^

#### Pre-service education and in-service training and support on AMR, appropriate antimicrobial use, and IPC

Although variations existed in health-care education systems and capacity across countries, the mission recommendations from the country reviews in Kazakhstan and Thailand included the need to cover AMR prevention and control competencies as part of training for the primary care workforce and standardise AMR-related academic modules across undergraduate and postgraduate health education.

The missions identified that once health workers were in service, continued support and regular training were important to help to translate theory into practice, introduce and regularly reinforce up-to-date IPC and AMS practices and treatment guidelines, and orient and integrate new health workers to ensure quality of care. Continued professional development frameworks and requirements varied across countries, although the relicensing process that health-care professionals commonly undertake at regular intervals could be an entry point for introducing and extending the scope of AMR-related in-service training, such as was recommended in Thailand. Community health worker programmes, including training requirements, also varied between countries, and strategies such as refresher training and supportive supervision were recommended to be applied in different contexts.

#### Build competence in IPC and appropriate antimicrobial use through improving skill-mix

Integrated workforce capacity in IPC and AMS was identified as an area requiring further support and strengthening in all three countries. In Kazakhstan, Sweden, and Thailand, integrated care was provided by multiprofessional teams, often consisting of doctors, nurses, and pharmacists at primary care facilities, and these teams usually had personnel with specific responsibility for IPC or AMS.

All three countries recognised the importance of IPC in preventing the acquisition and spread of AMR in health-care settings and ensuring overall quality of care, as reflected in the existence of national policies and observed implementation of interventions at the facility level. In Thailand, the Healthcare Accreditation Institute includes IPC as a key condition for accreditation of health-care institutions.^33^ Key gaps included monitoring of IPC and AMS interventions and data at the primary care level.

The experiences of all three countries indicated that a multidisciplinary team model can support better integration of IPC and AMS functions in primary care and can strengthen patient-centred models of care within the context of resource constraints. For example, ensuring that nurses staffing primary care facilities and long-term care facilities have a range of core AMR competencies, including in IPC and AMS.

In Kazakhstan, the mission recommended strengthening competencies at primary care facilities to include AMS, whether by having relevant skilled professionals on staff or by ensuring that primary care facilities had access to such support through referrals to and consultations with higher levels of care. In Sweden, there was a recognised need to strengthen AMS and IPC curricula for pre-service and in-service training for the primary care workforce.

## Discussion

The priority interventions identified through the country reviews align with the available evidence on specific policies and interventions to improve PHC, such as on education and motivation of the workforce,^34^ skill substitution and use of multidisciplinary teams, and promotion of health literacy;^35^^,^^36^ as well as on policies that can reduce inappropriate antibiotic use^27^ while strengthening access to essential antibiotics. GBD 2021 Antimicrobial Resistance Collaborators estimated that up to 92 million AMR-associated deaths could be averted by 2050 with improved quality of care for infectious syndromes and access to antibiotics.^2^

Primary care workers, including community health workers, should thus be equipped with the capabilities to provide quality care by preventing and treating infections appropriately, including appropriate prescribing of antibiotics within national frameworks. This aim can be achieved through comprehensive competency-based pre-service education and by providing ongoing support through in-service training and support in practice. WHO has defined general core competencies on AMR for health workers^37^, ^38^, ^39^ and more specific learning competencies for AMR in medical education^40^ and for IPC.^41^

In-service training is diverse across settings and should be defined depending on country health education, licensing and relicensing requirements, regulation systems, and health workers’ expectations. Available evidence, although of limited generalisability,^42^ suggests that education and training in themselves, without post-training support, are insufficient to improve service quality.^43^ Therefore, in-service support for primary care workers should also be prioritised, including for community health workers who are often on the frontlines of providing care for underserved communities. This support might take the form of practical tools and job aids, such as decision trees and checklists, and mentoring and supportive supervision wherever possible.

Because shortages and low retention rates of qualified health personnel in primary care facilities are common challenges in many countries, particularly in rural and remote areas, a skill-mix approach might be one that can ensure that primary care facilities have personnel with the needed capabilities on staff to prevent, diagnose, and appropriately treat infections. Although the evidence on the impact of multidisciplinary teams, also known as interprofessional teams, on patient outcomes is mixed, the experiences of the three countries suggest that integrated IPC and AMS functions through the skill-mix approach could be an important strategy to comprehensively address AMR in primary care.^44^, ^45^, ^46^, ^47^ Professional associations have a role to play in mentoring, supporting, and advocating for their members and colleagues to be able to perform IPC and AMS functions, as noted for all three countries.

Implementing AMS policies are key to improving appropriate antimicrobial use^7^ and are also important in improving quality of care and patient outcomes in PHC-oriented health systems. AMS programmes will look different in various countries, depending on the cultural context and whether incentives or regulations could be perceived to have more impact. Nevertheless, well-defined parameters and indicators can help to translate policy into practice for health-care providers and managers. Community pharmacists should be integrated into AMS programmes wherever possible because they are often the first point of contact for people with common symptoms and play a crucial role in counselling, referral, and appropriate dispensing of antibiotics.

Concurrently, collecting and using reliable and up-to-date diagnosis and prescription data through electronic health information systems can not only support implementation and monitoring of AMS programmes and AMR-relevant interventions but also strengthen quality and continuity of care by helping primary care teams to track patient diagnosis and prescribing history and outcomes. Some emerging areas of work that could be of interest to many countries include engaging private health-care providers in data sharing and use and exploration of how new technologies, such as artificial intelligence, can support the implementation of AMS policies and adherence to treatment guidelines through clinical decision support systems. Issues around data governance will need to be addressed more widely to enable expanded collaboration with both public and private health-care providers in this regard.

The country reviews reinforced that access to essential medicines is a building block of a functioning PHC-oriented health system, allowing primary care workers to provide effective treatment at first point of contact and reducing the burden on secondary and tertiary care facilities. Insufficient access to affordable, safe, and quality-assured essential antibiotics could lead to infection spread or suboptimal use, driving resistance selection and development.^48^ Affordability issues could also lead to inappropriate use of antibiotic practices. Inadequate access to essential antibiotics is a recognised problem in many LMICs,^7^ although even high-income countries can encounter shortages or inequitable access.^49^

Overall, the priority AMR interventions for primary care and the community identified through these reviews are consistent with evidence showing that global targets on AMR are unlikely to be met without stronger infection prevention measures, improved treatment of infections, and enhanced access to available essential antibiotics, in addition to drug development, particularly in LMICs.^2^ A 2024 modelling study estimated that 7·8% of AMR-associated mortality in LMICs could be prevented by IPC interventions, 5·7% by ensuring adequate WASH, and 4·2% through vaccination interventions,^50^ and these estimates are supported by further evidence on the impact of vaccines on reducing the incidence of infections.^51^ These data highlight a clear link between quality primary care services, including preventive public health interventions, and being able to reduce the number of infections and overuse or inappropriate use of antimicrobials. Therefore, essential services such as safe WASH; transport and logistics networks; and national immunisation programmes should be funded and implemented together, not separately, as part of a people-centred PHC approach, involving stakeholders across different sectors.

The country reviews described in this paper provide important qualitative and country-specific information supporting the PHC approach and highlight the importance of integrating AMR-relevant interventions within health systems. Notably, the eight AMR priority interventions identified in the WHO policy brief span all three components of the PHC approach while also cascading across multiple health system functions. That is, the interventions span system functions or levers of governance, workforce, financing, service delivery, information systems, and medicines, reinforcing that addressing AMR in primary care and the community requires a whole-of-system perspective rather than isolated or vertical actions. This finding emphasises that the PHC approach provides an essential framework to consider AMR-relevant interventions and that AMR and PHC-strengthening efforts are not a discrete technical exercise but should be an integrated effort embedded within broader health system design, stewardship, and financing arrangements.

Each of the three countries reviewed in this paper differed in terms of both income level and health system structure.^15^ The application of the scoping tool in each country provided insights into shared themes but, within the shared themes, different country experiences were observed, making clear that the design of AMR-relevant interventions should consider social, cultural, and contextual differences that affect the uptake and effectiveness of interventions. For instance, the type of incentives that are likely to bring about appropriate behaviour change in prescribers and communities and the nature of training and support provided to primary care workers will vary depending on the country context. The country reviews also emphasised the need to involve a broader and more diverse range of stakeholder groups when planning and implementing AMR interventions in primary care and the community. In particular, community engagement and participation in governance mechanisms can help to ensure that health services remain equitable and responsive to needs and that healthy behaviours in the community are encouraged and sustained.

The analysis of country reviews in this Health Policy paper has some limitations. Only three countries were involved in the refinement process, thereby restricting the generalisability of the findings for other countries, especially in lower-income settings. Public primary care facilities visited during site visits were usually selected on the basis of convenience and were not representative. Not all the technical areas included in the AMR and PHC scoping tool and WHO’s PCA-AMR scoping tool were selected for review by the countries, and thus, not all areas of the tool were tested in the countries (table 1). Other countries’ priorities and areas of intersecting AMR and PHC focus could ultimately differ. Additional reviews are planned in other countries, which could further show how the scoping tool can be used and adapted in other countries.

The country reviews highlighted that considerable work relevant to AMR outcomes is ongoing in primary care and the community, although this work is not always labelled specifically as AMR or explicitly referenced in national action plans on AMR. The reviews showed the applicability of the AMR and PHC scoping tool across three different health system contexts and its usefulness in identifying ongoing activities on AMR in primary care and communities. They also showed how the scoping tool could be used to identify gaps and practical opportunities for integrating AMR interventions into PHC-oriented health systems. The updated Global Action Plan on AMR 2026–36 highlights the importance of integrating AMR-related interventions into health system-strengthening efforts.^13^ Notably, national AMR programmes should work with health system planners to integrate AMR-related interventions into PHC-oriented health systems, strengthening them to drive progress in countries towards UHC while achieving global targets on AMR. As PHC continues to remain a priority for national health systems, efforts made to strengthen PHC-oriented health systems can be expected to yield broad positive health outcomes, including on AMR.

**Search strategy and selection criteria**

References for the desk reviews described in this Health Policy paper were identified through searches of Google Scholar between April and December 2024 using a combination of terms, including “antimicrobial resistance”, “antimicrobial stewardship”, “primary health care”, “primary care”, “infection prevention and control”, and “access to essential medicines” and relevant country names, with no date or language restrictions applied given the low volume of literature expected on each topic for each country. These references were triangulated with information from national policy documents and government reports, identified through national government and ministry websites, WHO regional and country office repositories, and WHO databases. Country-level data not available in the public domain were requested directly from national counterparts and relevant WHO country and regional offices. Wherever documented evidence was insufficient, structured or semi-structured input was solicited directly from national focal points leading AMR and PHC work in each country to supplement or clarify the desk-based findings. Materials reviewed were primarily in English, with some being in Thai, Russian, or Swedish, reflecting the official or working languages of the respective countries. Non-English language documents were translated and reviewed by fluent team members. The final selection was based on credibility and relevance to the topics covered in this Health Policy paper.

## Declaration of interests

SWYC is engaged as a consultant with WHO, and this manuscript was developed as part of the overall consultancy agreement with WHO. All other authors declare no competing interests.
