# Supplementary material for: Refinement of a scoping tool to tackle antimicrobial resistance via a primary health care approach: insights from Kazakhstan, Sweden, and Thailand
Source: Lancet Prim Care. 2026 Sep;2(9):None. doi: 10.1016/j.lanprc.2026.100211 (PMC13642074; doi:10.1016/j.lanprc.2026.100211)
Supplement: Supplementary file 1 [file mmc1.pdf]

THE LANCET
Primary Care

Supplementary appendix

This appendix formed part of the original submission and has been peer reviewed.
We post it as supplied by the authors.

Supplement to: Chong SWY, Paulin S, Khalid F, et al. Refinement of a scoping tool to tackle antimicrobial resistance via a primary health care approach: insights from Kazakhstan, Sweden, and Thailand. *Lancet Prim Care* 2026. https://doi.org/10.1016/j.lanprc.2026.100211

Equitable Partnership Declaration

If any questions do not apply to your study, please indicate "N/A" for "not applicable.
For more information on how to complete this form see the Information for Authors document.

Researcher considerations

1. Please detail the involvement that researchers who are based in the country or countries of study had during a) study design; b) clinical study processes, such as processing blood samples, prescribing medication, or patient recruitment; c) data interpretation; and d) manuscript preparation, commenting on all aspects. If they were not involved in any of these aspects, please explain why.

   This should include a thorough description of their leadership roles in the study. Are local researchers named in the author list or the acknowledgements, or are they not mentioned at all (and, if not, why)? Please also describe the involvement of early career researchers based in the location of the study. Some of this information might be repeated from the Contributors section in the manuscript. Note: we adhere to ICMJE authorship criteria for naming authors on a paper.

**a) Study design:**<br>The co-authors based in the countries of study (Kazakhstan, Sweden, and Thailand) all led the process of identifying the focus areas for research in each country. They also led and participated in the process of data collection through a desktop review and by leading and facilitating site visits and group discussions.
**b) Clinical study processes:**<br>N/A
**c) Data interpretation:**<br>The co-authors based in the countries of study all participated in the process of analysing and validating observational and qualitative data gathered through desktop review, site visits and stakeholder group discussions.
**d) Manuscript preparation:**<br>The co-authors based in the countries of study all participated in reviewing and providing inputs into the manuscript.

2. How was funding used to remunerate and enhance the skills of researchers in the countries of study? And how was funding used to improve research infrastructure at the study sites?

   Potentially effective investments into long-term skills and opportunities within local institutions could include training or mentorship in analytical techniques and manuscript writing, opportunities to lead all or specific aspects of the study, financial remuneration rather than requiring volunteers, and other professional development and educational opportunities.

Improvements to research infrastructure could include funding extended trial designs (eg, platform trials), establishment of long-term contracts for research staff, building research facilities, and setting up local control of funding allocation.

**Skills:**<br>Soft training in workshop design and facilitation; data collection through desk review, and opportunities to lead site visits and group discussions.
**Research infrastructure:**<br>N/A

3. How did you safeguard the researchers who implemented the study?

Please describe how you guaranteed safe working conditions for study staff, including provision of appropriate personal protective equipment, protection from violence, and prevention of overworking.

The researchers that were involved did so in the capacity of their institution which provided safe working environments. Data collection and facilitation tasks during the mission were equally distributed to ensure prevention of overworking.

Benefits to the communities and regions of study

4. How does the study address the research and policy priorities of its location?

How were the local priorities determined and then used to inform the research question? Who decided which priorities to take forward? Which elements of the study address those priorities?

Local priorities and focus areas for research were identified through a process of engaging with national focal points, usually based within a national health agency, led and facilitated by the co-authors in the countries of study. This was further supported by an inclusive stakeholder consultation process, where workshop participants were all local health care personnel, working in and with communities in the countries of interest.

5. How will research products be shared in the community of study?

For instance, will you be providing written or oral layperson summaries for non-academic information sharing? Will study data be made available to institutions in the region(s) of study? The Lancet Global Health *encourages authors to translate the summary (abstract) into relevant languages after paper editing; do you intend to translate your summary?*

Some research products have already been published for wider dissemination to a non-academic audience. For example, mission reports for each country of study have been jointly published by WHO and national health agencies, translated into the local language for both Thailand and Kazakhstan, and news/media reports have provided some key points arising from the research, targeted at the general public. The WHO policy brief that arose from this piece of research will be published in all other official UN languages.

6. How were individuals, communities, and environments protected from harm?

a) *How did you ensure that sensitive patient data were handled safely and respectfully? Was there any potential for stigma or discrimination against participants arising from any of the procedures or outcomes of the study?*

No patient data was collected for this study.

b) *Might any of the tests be experienced as invasive or culturally insensitive?*

No tests were undertaken for this study.

c) *How did you determine that work was sensitive to traditions, restrictions, and considerations of all cultural and religious groups in the study population?*

Site visits undertaken were led and facilitated by national focal points and co-authors based in the countries of study, thus ensuring that the work was sensitive to traditions, restrictions, and considerations of local communities in each country.

d) *Were biowaste and radioactive waste disposed of in accordance with local laws?*

No biowaste/radioactive waste was generated.

e) *Were any structures built that would have impacted members of the community or the environment (such as handwashing facilities in a public space)? If so, how did you ensure that you had appropriate community buy-in?*

N/A

f) *How might the study have impacted existing health-care resources (such as staff workloads, use of equipment that is typically employed elsewhere, or reallocation of public funds)?*

The study involved site visits to health facilities and 2 or 3-day workshops. Health care workers at the facilities visited took time away from their normal duties to participate in discussions and answer our questions – these visits typically lasted 1-3 hours at most to limit the disruption. Participants in country workshops took time away from their normal work to participate in workshops. Where possible, workshops were planned so that participants would not need to take excessive time away from their normal work, and in all cases, participants were nominated to participate by national/subnational health authorities, who released them from their duties for the duration of participation.

7. Confirm that local ethics review was sought, and please provide the approval number. If not sought, please explain why.

Not sought – not applicable.

Secondary analyses

8. Have the data analysed in your study been extracted from another source, such as a national survey, rather than being directly collected by the authors of this paper?

Yes

If the authors of this paper were not involved in data collection, how were the findings interpreted with sufficient contextual knowledge?
The Lancet Global Health *believe contextual understanding is crucial for informed data analysis and interpretation*.

All findings provided in this study were verified and validated with local stakeholders, and data was triangulated to support the conclusions drawn.

---

9. Please provide the title (eg, Dr/Prof, Mr/Mrs/Ms/Mx), name, and email address of an author who can be contacted about this statement.

**Name:** Dr. Nienke Bruinsma<br>**Email:** Bruinsma@who.int

10. Finally, please provide the title and name of an author from one country of study who has seen and approved this form.

**Name:** Stephan Stenmark<br>**Email:** stephan.stenmark@folkhalsomyndigheten.se
